# Perceptions of Rheumatologists on Diagnosis of Psoriatic Arthritis in China

**DOI:** 10.3389/fimmu.2021.733708

**Published:** 2021-12-03

**Authors:** Miao Chen, Hua Zhang, Zhiyong Chen, Sheng-Ming Dai

**Affiliations:** Department of Rheumatology & Immunology, Shanghai Jiao Tong University Affiliated Sixth People’s Hospital, Shanghai, China

**Keywords:** psoriatic arthritis, early diagnosis, rheumatologists’ perceptions, questionnaire, survey

## Abstract

**Objective:**

High prevalence of undiagnosed psoriatic arthritis (PsA) and prolonged diagnostic delay are key troubles in the appropriate management of PsA. To analyze the possible causes for this phenomenon, a web-based nationwide survey was conducted to investigate rheumatologists’ perceptions on PsA diagnosis in China.

**Methods:**

The electronic questionnaire consisting of 38 questions were designed by an expert panel and distributed with the online survey tool Sojump, which is a professional online survey platform. The completed questionnaires by real-name rheumatologists were collected.

**Results:**

A total of 1594 valid questionnaires were included. More than half of Chinese rheumatologists reported it was challenging to make a diagnosis of PsA. The four major challenges were “Difficulties in identification of atypical or hidden psoriasis”, “Absence of diagnostic biomarkers”, “No active self-report of history or family history of psoriasis” and “Various musculoskeletal manifestations”. In diagnosing PsA, minor participants had incorrect knowledge of inflammatory arthropathy (13.7%), acute phase reactant (23.8%), and rheumatoid factor (28.7%). There were no significant differences in the knowledge of PsA and practice habits in diagnosing PsA between modern western medicine (WM)- and traditional Chinese medicine (TCM)-rheumatologists. The part-time rheumatologists were not as good as full-time rheumatologists in diagnosing PsA.

**Conclusions:**

About three quarters of Chinese rheumatologists are familiar with the elements in PsA diagnosis and have good practice habits in diagnosing PsA. Four main challenges in making PsA diagnosis are revealed. There was no significant difference in the knowledge of PsA between WM- and TCM-rheumatologists.

## Introduction

Psoriatic arthritis (PsA) is a form of chronic inflammatory arthritis associated with psoriasis. It is characterized by a wide clinical spectrum with diverse affected organs and tissues, including peripheral and axial joints, nail, skin and entheses (1). In the majority of PsA patients, nail and skin lesions antedate musculoskeletal manifestations (2). However, approximately 20% of PsA patients develop arthritis prior to the onset of psoriasis, and 15% of them show psoriasis and arthritis almost simultaneously (3). The wide clinical spectrum and variable disease course bring challenge to early detection of PsA. In addition, the absence of serum diagnostic biomarkers and no specific hallmark of imaging examinations are also obstacles to the early diagnosis of PsA (4, 5). As an erosive disease, delayed diagnosis of PsA leads to a poor outcome of the patients. There is plenty of evidence that irreversible joint damage can occur in the first year of disease course (6–8), and even a six-month delay in diagnosis leads to radiographic damage (9). Moreover, PsA is associated with various comorbidities like hypertensions, type 2 diabetes, depression, and inflammatory bowel disease, which brings to huge burdens to society (10, 11). Therefore, early detection of PsA is essential to reduce the burden of disease.

High prevalence of undiagnosed PsA and prolonged diagnostic delay are still key troubles in the appropriate management of PsA throughout the world (5). According to a systematic review, 15.5% of patients with psoriasis at dermatologic centers had undiagnosed PsA (12). A recent population-based study demonstrated that more than half of PsA patients had a delay in diagnosis of two or more years (13). The insufficient knowledge on PsA in rheumatologists may also lead to the misdiagnosis of PsA. According to the Survey of Chinese Rheumatologists in 2014, there were only 4515 specialized rheumatologists in China taking care of the tremendous number of patients with rheumatic diseases (14). In order to deal with the shortage of full-time rheumatologists in China, there are many physicians who work as part-time rheumatologists, such as nephrologists-rheumatologists, hematologists-rheumatologists, and endocrinologists-rheumatologists. In addition, there are many rheumatologists with traditional Chinese medicine (TCM) in China. We conducted a web-based nationwide survey to analyze the possible causes for underdiagnosed PsA, and to compare the knowledge of PsA and practice habits in diagnosing PsA among different rheumatologists in China.

## Materials and Methods

### Questionnaire Design

The electronic questionnaires titled “The current status of psoriatic arthritis diagnosis in China” were designed by an expert panel consisting of six rheumatologists. The questions were designed according to the problems they encountered during clinical practice in PsA diagnosis. The final electronic questionnaire was composed of 38 questions in two parts consisting of basic information of respondents in part 1 and perceptions on PsA diagnosis in part 2. After a short introduction of this survey, the electronic consent to survey participation was distributed in the first page. The following approaches were used to protect unauthorized access. Before signing the consent with real name was submitted, the body of the questionnaire couldn’t be accessed. The informed consent must be given before moving on to the body of the questionnaire. The electronic questionnaires could not be revised and collected through the links. To ensure the eligibility of participants for this survey, all the participants were required to supply with their real names and real hospital names where they are employed to guarantee the eligibility of participants. Their real names and real hospital names were further verified by the leaders of local rheumatology associations to judge the participants’ eligibility. If unauthorized access occurred, the unqualified participants will be ruled out from the analysis. The part of perceptions on PsA diagnosis included five domains as “The challenges in making PsA diagnosis”, “Knowledge of Musculoskeletal manifestations”, “Cognizance of psoriatic skin and nail lesions”, “Laboratory tests” and “Clinical practice habits in dealing with key clues of PsA”. Except for the names of participants and their employers/hospitals which were fill-in-the-blank questions, all the other questions were presented in a choice format as sole choice or multiple choices. There were 2 to 9 choices in different questions. For some questions, if rheumatologists did not agree with the listed choices, they could select the choice of “others” and write their answers. The questionnaire could not be submitted until all the questions had been completed. The respondents could review and change the answers before the questionnaire were submitted.

Before the questionnaires were distributed online, pilot tests were conducted among 20 rheumatologists for five times. The questionnaire was revised according to the concerns arising in the pilot tests, including modifying sentences that were prone to ambiguity; adding alternative answers to include various possibilities and marking the words in red which were needed to be emphasized. After five times of pilot tests, the questionnaire was finally determined.

### Samples and Survey Method

In-service rheumatologists across China mainland who could read Chinese were eligible for this survey. Doctors don’t work as rheumatologists were excluded. WeChat is a free application developed by Tencent, Inc., and has become the most widely and frequently used social networking platform in China (15). According to the latest data from Tencent, Inc., the number of active monthly customers reached 1.24 billion in the first quarter of 2021 (16). This tremendous number of users provides WeChat potential application in web-based survey. In this survey, the electronic questionnaires were produced, distributed, and collected with the online survey tool Sojump (http://www.sojump.com) (17), which is a professional online survey platform. Snowball sampling *via* the WeChat contacts network was adopted in this survey. The survey links were initially sent to all the thirty-one leaders of local (province or city) rheumatology associations. They were asked to spread the survey links on their WeChat contact network including rheumatologists personal accounts and WeChat groups composed of rheumatologists and encourage them to participate in the survey. Every receiver was encouraged to spread the questionnaire link to their own departments. The questionnaires were completed voluntarily without any compensation. To prevent from duplicate responses, only one submission is allowed per WeChat account as well as per mobile device. In China, a mobile phone number must be registered with a person’s ID card, and WeChat account must be linked to a real mobile phone number. Their real names and real hospital names were verified by the leaders of local rheumatology associations to further judge the participants’ eligibility. This study is a cross-sectional survey without follow-up. The survey links were open from March 20, 2021 to May 10, 2021. The activation period of the survey was one month prior to the survey links were open. During this period, the pilot tests were conducted and the leaders of local rheumatology associations were requested to call for rheumatologists in their provinces or cities to participate in this survey.

The study was approved by the Ethics Committee of Shanghai Jiao Tong University Affiliated Sixth People’s Hospital, Shanghai, China. Electronic informed consent was obtained from each participant.

### Statistical Analyses

The invalid questionnaires from non-rheumatologists and the duplicate questionnaires were eliminated. The data of questionnaire were exported from Sojump. As we set that the questionnaire could not be submitted until all the questions had been completed, so there are no missing data. The categorical variables were presented as numbers or percentages. Chi-square tests were used to compare unordered categorical variables. Wilcoxon rank sum tests were used to compare ordinal categorical variables. Kendall tau rank correlations were used to investigate the correlation of ordinal categorical variables. P < 0.05 was considered significant. All the analyses were performed using SAS 8.0 (SAS Institute, Cary, NC, USA).

## Results

**Figure 1:**
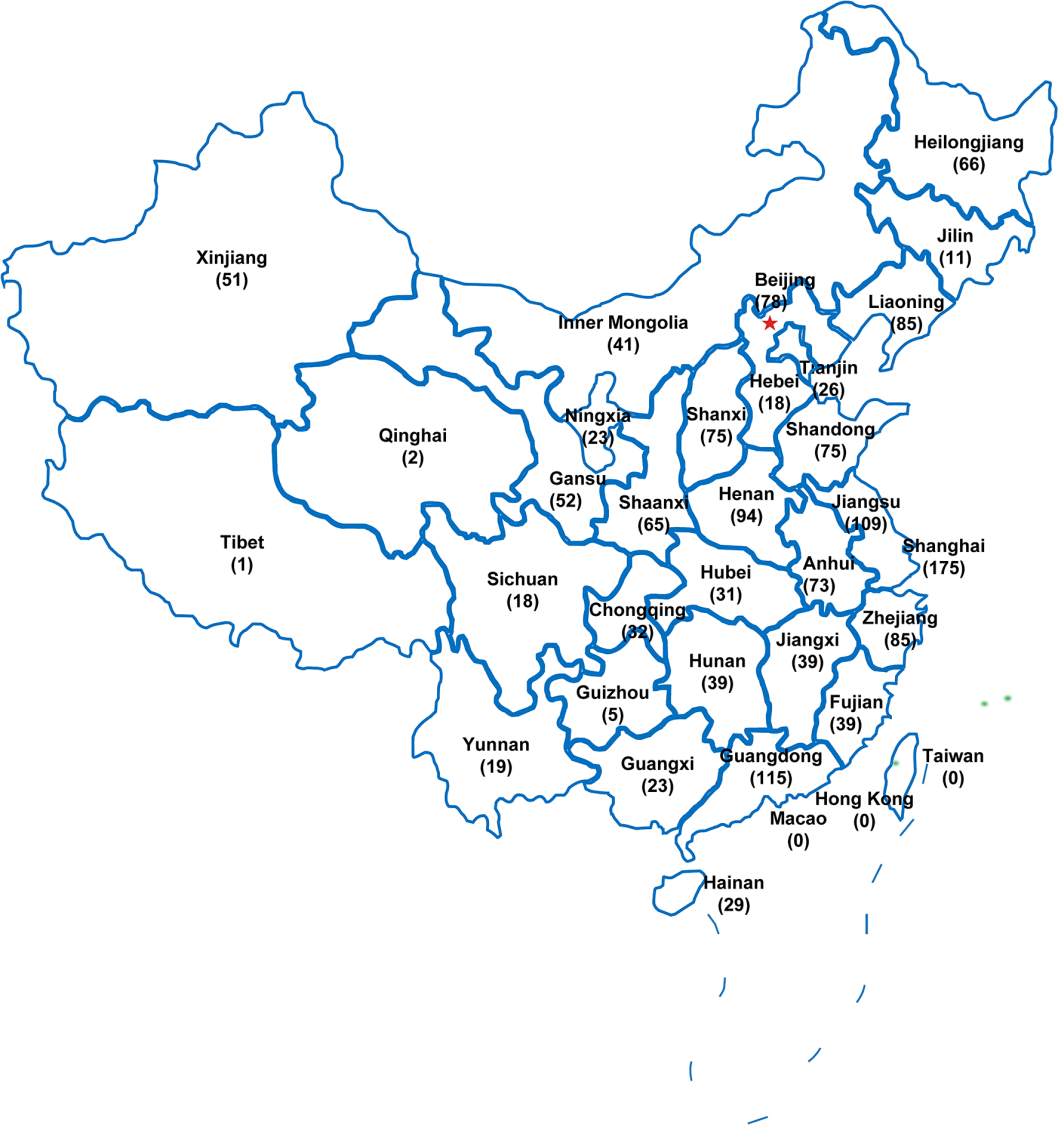
The geographical distribution of the subjects recruited in this survey (n=1594). The number of respondents in every administrative region were shown in brackets. The pentagram indicates the capital.

A total of 1652 electronic questionnaires were collected. After excluding of the questionnaires from non-rheumatologists and the duplicate questionnaires, a total of 1594 valid questionnaires were included in the analysis. There are 31 administrative regions in China mainland, including 22 provinces, 5 autonomous regions and 4 municipalities, and the respondents located widely in every province and region (**Figure 1**).

### Characteristics of the Respondents

As shown in **Table 1**, nearly four-fifths of respondents were western medicine (WM)-rheumatologists. Most of respondents worked in tertiary hospitals (i.e., Chinese grade 3A hospitals) (92.0%) and were full-time rheumatologists (81.3%). The proportions of respondents working in tertiary hospitals and full-time rheumatologists were significantly higher among WM-rheumatologists than that among TCM-rheumatologists (both P<0.01). More than seventy percent of respondents had working experience as rheumatologists over six years. Nearly four-fifths of rheumatologists (79.0%) established ≤3 new diagnoses of PsA every month among those first-time referred patients. The number of new cases of PsA per month were positive related with working experience as rheumatologists (P<0.001) (**Supplementary Figure 1**).

**Table 1:** Characteristics of respondents.

Characteristics	Western Medicine- Rheumatologistsn = 1256 (n, %)	Traditional Chinese Medicine- Rheumatologists n = 338 (n, %)	Total n = 1594 (n, %)
**Age (years)**			
<30	98 (7.80)	19 (5.62)	117 (7.34)
30-40	498 (39.65)	147 (43.49)	645 (40.46)
41-50	391 (31.13)	104 (30.77)	495 (31.05)
>50	269 (21.42)	68 (20.12)	337 (21.14)
**Levels of hospitals**			
Tertiary	1179 (93.87)	288 (85.21)	1467 (92.03)
Secondary	77 (6.13)	50 (14.79)	127 (7.97)
**Specialty**			
Full-time rheumatologists	1040 (82.80)	256 (75.74)	1296 (81.30)
Part-time rheumatologists	216 (17.20)	82 (24.26)	298 (18.70)
**Working experience as rheumatologists (years)**			
≤5	295 (23.49)	78 (23.08)	373 (23.40)
6-10	304 (24.20)	87 (25.74)	391 (24.53)
11-20	396 (31.53)	106 (31.36)	502 (31.49)
>20	261 (20.78)	67 (19.82)	328 (20.58)
**Number of newly diagnosed PsA patients per month**			
<1	436 (34.71)	141 (41.72)	577 (36.20)
1-3	543 (43.23)	139 (41.12)	682 (42.79)
4-5	173 (13.77)	45 (13.31)	218 (13.68)
6-10	82 (6.53)	8 (2.37)	90 (5.65)
11-20	16 (1.27)	3 (0.89)	19 (1.19)
>20	6 (0.48)	2 (5.92)	8 (0.50)

PsA, Psoriatic arthritis.

### The Challenges in Making PsA Diagnosis

**Figure 2:**
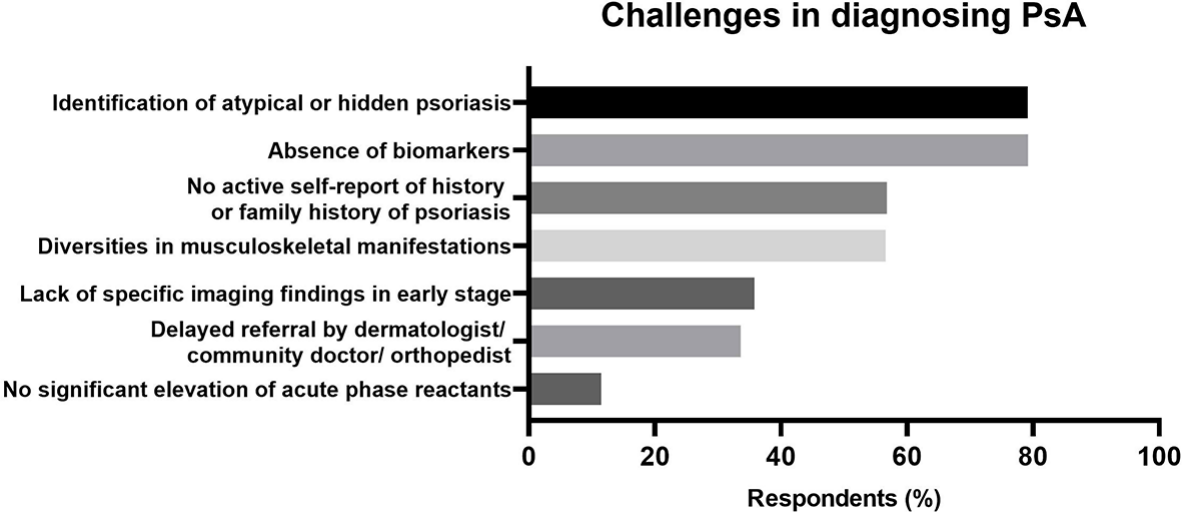
Difficulties in diagnosing Psoriatic Arthritis (PsA) reported by rheumatologists. The challenging items were shown in the vertical axes. The percentages of positive responses were shown in the horizontal axes.

**Figure 3:**
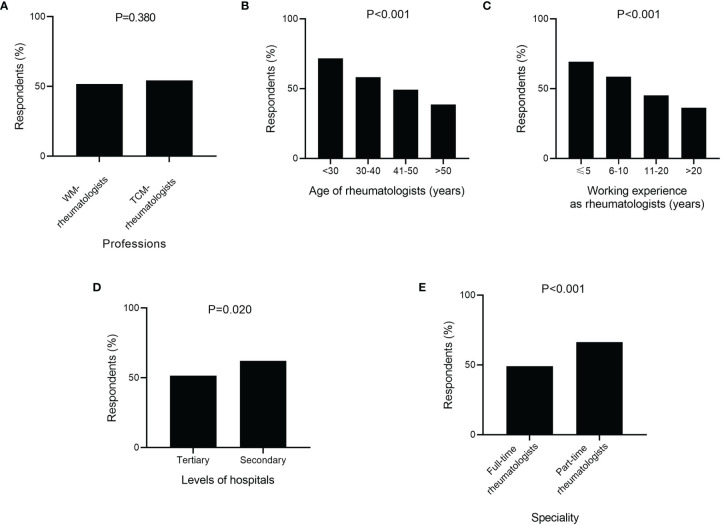
Stratified analysis of respondents with challenges in diagnosing psoriatic arthritis (PsA) by medical training system **(A)**, age of rheumatologists **(B)**, working experience as rheumatologists **(C)**, levels of hospitals **(D)**, and speciality of respondents **(E)**. **(A)** WM, Western Medicine; TCM, Traditional Chinese Medicine.

Overall, approximately half of respondents (52.3%) reported it was difficult to make a diagnosis of PsA. There were four main challenges in making diagnosis of PsA (**Figure 2**). Nearly four fifth of rheumatologists regarded “Identification of atypical or hidden psoriasis” (79.2%) and “Absence of diagnostic biomarkers” (79.2%) as difficulties in making diagnosis of PsA, while more than half of rheumatologists thought “No active self-report of history or family history of psoriasis” (56.8%) and “Various musculoskeletal manifestations” (56.6%) were barriers to a correct diagnosis of PsA. Additionally, the lack of specific imaging findings in early stage and delayed referral were also regarded as challenges in making in-time diagnosis of PsA by about forty percent of rheumatologists. The proportions of rheumatologists who reported it was difficult to make PsA diagnosis were higher among ones with younger age, less working experience, working in secondary hospitals and part-time rheumatologists (**Figure 3**), but were not different between the WM- and TCM-rheumatologists.

### Perceptions of Elements in PsA Diagnosis

#### Knowledge of Musculoskeletal Manifestations

**Figure 4:**
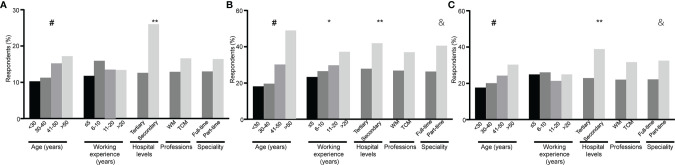
Stratified analysis of respondents with misconception in diagnosing of psoriatic arthritis (PsA) by age of rheumatologists, working experience as rheumatologists, levels of hospitals, medical training system and speciality of respondents. The percentages of respondents who answered incorrectly (“Yes”) to the following questions are shown. **(A)** Do you think the diagnosis of PsA could be made once psoriasis patients presented arthralgia? **(B)** Do you think the diagnosis of PsA could be ruled out when patients with psoriasis had positive rheumatoid factors (RF) or anti-cyclic citrullinate peptide antibodies (ACPA)? **(C)** Do you think the elevation of erythrocyte sedimentation rate (ESR) or C-reactive protein (CRP) is a prerequisite for the diagnosis of PsA? ^#^ represents the percentages of respondents significantly differ among age groups (P < 0.01). * represents the percentages of respondents significantly differ among working experience groups (P < 0.01). ** represents the percentages of respondents significantly differ between levels of hospitals (P < 0.01). & represents the percentages of respondents significantly differ between speciality of rheumatologists (P < 0.01). WM, Western Medicine; TCM, Traditional Chinese Medicine.

Only a small part of rheumatologists (13.7%) mistakenly believed that the diagnosis of PsA could be made once psoriasis patients presented arthralgia. The incidences of this misconception significantly increased with the age of rheumatologists (P=0.0041) and were higher among rheumatologists from secondary hospitals than those from tertiary hospitals (P<0.001) but did not differ between the WM- and TCM-rheumatologists (**Figure 4A**).

**Figure 5:**
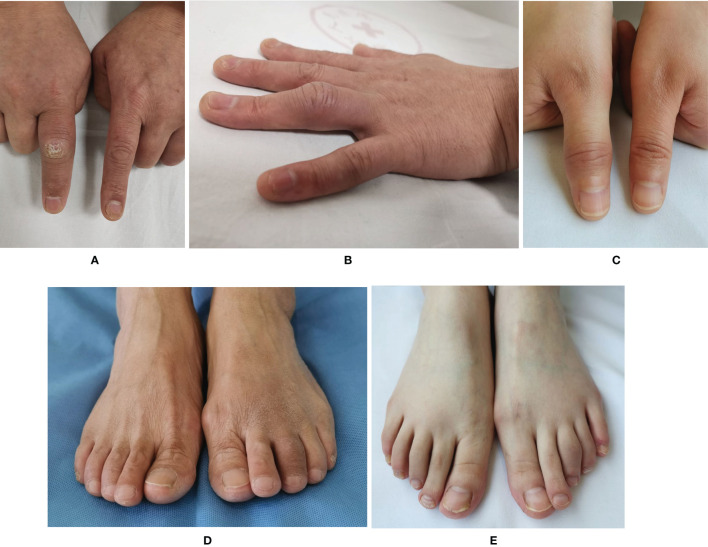
Different manifestations of dactylitis. **(A)** Typical dactylitis in the index finger of right hand. **(B)** Atypical dactylitis of the ring finger with marked swelling of the proximal interphalangeal joints but without metacarpophalangeal and distal interphalangeal joints involved. **(C)** Atypical dactylitis in the thumb of the right hand with marked swelling of the interphalangeal joint of thumb but without metacarpophalangeal joint involved. **(D)** Typical dactylitis in the third toe of left foot with whole toe swelling. **(E)** Atypical dactylitis in the third toe of left foot with marked swelling of the proximal interphalangeal joint of third toe but without metatarsophalangeal and distal interphalangeal joints involved.

The attitudes of rheumatologists on the role of PsA specific manifestations in its diagnosis, such as distal interdigital arthritis and dactylitis, were assessed. When a patient had a single distal interphalangeal (DIP) joint swelling, more than ninety percent of respondents (93.2%) considered PsA diagnosis. There were 53.1% of respondents who agreed that dactylitis was a hallmark of PsA in its diagnosis. In regard of definition of dactylitis, about one fifth of rheumatologists (19.6%) believed that only a whole swollen digit could be defined as dactylitis or sausage digit (**Figures 5A, D**), while 42.7% held the opinions that the marked swelling of a proximal interphalangeal (PIP) joint with juxta-articular soft tissue but without swelling of metacarpophalangeal (MCP) or DIP joint (**Figures 5B, C, E**) were also termed dactylitis. There was no difference in the attitudes of rheumatologists of WM and TCM, as well as different ages, levels of hospitals, and specialties to the role of distal interdigital arthritis and dactylitis in PsA diagnosis.

#### Cognizance of Psoriatic Skin and Nail Lesions

In identification of psoriatic skin and nail lesions, 96.5% and 88.2% of the respondents relied on dermatologists’ judgements, respectively. Almost all the participants (96.4%) knew nail pitting, but cognition rates of other types of psoriatic nail lesions were not so good: nail plate crumbling (70.4%), subungual hyperkeratosis (64.7%), onycholysis (57.4%), leukonychia (31.6%), oil-drop discoloration (27.5%), splinter hemorrhages (24.6%), and red spots in the lunula (18.2%). On the contrary, 27.5% of the respondents mistakenly considered nail discoloration as psoriatic nail lesions, and the incidence of mistakes increased with the age of rheumatologists.

#### Knowledge of Laboratory Tests

Nearly thirty percent of rheumatologists (28.7%) mistakenly believed that a diagnosis of PsA could be ruled out when patients with psoriasis had positive rheumatoid factor (RF) or anti-cyclic citrullinate peptide antibody (ACPA). In respect to the role of acute reactive reactants in PsA diagnosis, 23.8% of rheumatologists mistakenly believed that the elevations of acute phase reactants were a prerequisite for PsA diagnosis. The incidences of misconceptions on both of above two questions were higher among ones with older age, working in secondary hospitals and part-time rheumatologists, but were not different between WM- and TCW-rheumatologists (**Figures 4B, C**).

### Clinical Practice Habits in Dealing With Key Clues of PsA

In order to investigate the clinical practice habits of rheumatologists when dealing with key clues of PsA, a series of questions were designed. The questions were focused on query of psoriasis family history and examinations of skin and nail when patients presented certain related musculoskeletal manifestations including peripheral oligoarthritis, seronegative peripheral arthritis, asymmetric sacroiliitis, sacroiliitis with negative HLA-B27, and dactylitis (**Table 2**). Most of rheumatologists responded that they would carefully inquire about family history of psoriasis and check skin and nail psoriasis of patients, though the percentages varied in ages of rheumatologists, levels of hospitals, and full-time or part-time rheumatologists, but did not differ between the WM- and TCM-rheumatologists.

**Table 2:** Medical practice habits of rheumatologists in responding to key clues of PsA.

Questions	Q1 (Examination of nails and skin when a patient presents peripheral oligoarthritis)			Q2 (Examination of nails and skin when a patient manifests seronegative peripheral arthritis)			Q3 (Examination of nails and skin when a patient manifests asymmetric sacroiliitis)			Q4 (Examination of nails and skin when a patient manifests sacroiliitis with negative HLA-B27)			Q5 (Examination of nails and skin when a patient manifests dactylitis)			Q6 (Query of family history of psoriasis when a patient manifests dactylitis)		
	Yes (%)	No (%)	P value	Yes (%)	No (%)	P value	Yes (%)	No (%)	P value	Yes (%)	No (%)	P value	Yes (%)	No (%)	P value	Yes (%)	No (%)	P value
**Total**	84.2	15.8	NA	87.0	13.0	NA	82.3	17.7	NA	82.9	17.1	NA	91.1	8.9	NA	94.2	5.8	NA
**Age (years)**																		
<30	76.1	23.9	**0.002**	83.8	16.2	**0.003**	78.6	21.4	**0.015**	80.3	19.7	**0.048**	85.5	14.5	**0.001**	89.7	10.3	**0.015**
31-40	82.3	17.7		84.3	15.7		80.2	19.8		81.4	18.6		89.9	10.1		94	6.0	
41-50	86.7	13.3		88.9	11.1		83.8	16.2		83.4	16.6		91.1	8.9		93.7	6.3	
>50	86.9	13.1		90.2	9.8		85.5	14.5		86.1	13.9		95.3	4.7		96.7	3.3	
**Working experience as rheumatologists (years)**																		
≤5	77.7	22.3	**<0.001**	81.5	18.5	**<0.001**	78.8	21.2	**0.001**	79.6	20.4	**0.029**	88.2	11.8	**<0.001**	91.2	8.8	**<0.001**
6-10	83.9	16.1		86.4	13.6		78.8	21.2		82.4	17.6		89.5	10.5		92.6	7.4	
11-20	85.7	14.3		89.0	11.0		85.3	14.7		84.3	15.7		91.8	8.2		96.0	4.0	
>20	89.6	10.4		90.5	9.5		86.0	14.0		85.4	14.6		95.1	4.9		96.6	3.4	
**Levels of hospitals**																		
Tertiary	84.9	15.1	**0.012**	87.6	12.4	**0.010**	82.9	17.1	**0.039**	83.4	16.6	0.072	91.5	8.5	**0.030**	94.7	5.3	**0.003**
Secondary	76.4	23.6		79.5	20.5		75.6	24.4		77.2	22.8		85.8	14.2		88.2	11.8	
**Specialty**																		
Full-time rheumatologists	85.6	14.4	**0.002**	82.6	17.4	**0.001**	82.5	17.5	**0.006**	84.1	15.9	**0.010**	91.7	8.3	0.093	94.7	5.3	0.070
Part-time rheumatologists	78.2	21.8		72.6	27.4		75.5	23.2		77.9	22.1		88.6	11.4		91.9	8.1	
**Professions**																		
WM-rheumatologists	84.6	15.4	0.443	87.5	12.5	0.210	82.9	17.1	0.248	83.1	16.9	0.705	90.8	9.2	0.377	94.6	5.4	0.168
TCM-Rheumatologists *	82.8	17.2		84.9	15.1		80.2	19.8		82.2	17.8		92.3	7.7		92.6	7.4	

PsA, Psoriatic Arthritis; WM, Western Medicine; TCM, Traditional Chinese Medicine.

*Traditional Chinese Medicine rheumatologists and Integrated Chinese and Western Medicine rheumatologists were included.

Q1: When a patient presents peripheral oligoarthritis, would you carefully check their skin and nails? Q2: When a patient manifests seronegative peripheral arthritis, would you carefully check their skin and nails? Q3: When a patient manifests asymmetric sacroiliitis, would you carefully check their skin and nails? Q4: When a patient manifests sacroiliitis with negative HLA-B27, would you carefully check their skin and nails? Q5: When a patient manifests dactylitis, would you carefully check their skin and nails? Q6: When a patient manifests dactylitis, would you patiently ask if they have a family history of psoriasis?

P values below 0.05 are shown in bold.

## Discussion

This survey has a high sampling ratio of rheumatologists and covered all the thirty-one administrative regions in mainland China. About one third of Chinese rheumatologists participated in this survey (14). The relatively large sample size makes the research data more likely to represent the real perceptions of rheumatologists. Most of the respondents worked in tertiary hospitals and have worked for more than six years with relative more clinical experience. However, nearly 80% of rheumatologists established new diagnosis of PsA in no more than 4 first-referral patients every month, which might be attributed to the low prevalence of PsA, about 5.8% in Chinese patients with psorisis (18). In contrast, more than half of rheumatologists established new diagnosis of RA in more than ten first-referral patients every month in our present survey. This is consistent with the huge gap in the prevalence of RA and PsA. According to a population-based epidemiological investigation in China, the prevalence of RA was 0.28% while PsA was 0.02% (19). This far lower prevalence of PsA might be one of factors contributing to misconceptions of rheumatologists in PsA.

The challenges in PsA diagnosis were explored among rheumatologists. The proportions of rheumatologists, who reported it was challenging to make a diagnosis of PsA, varied in age, working years, levels of hospitals and specialties. The rheumatologists with older age and more working years were reasonably more confident and felt less difficulty in PsA diagnosis. “Identification of atypical or hidden psoriasis” and “No active self-report of history or family history of psoriasis” were the two main difficulties in diagnosing PsA. The main complaints of patients attending rheumatology clinic were musculoskeletal symptoms. As most of psoriasis patients are unaware of the internal relationship between musculoskeletal symptoms and skin lesions of psoriasis, they are focused on complaining about their arthritis symptom to rheumatologists instead of describing their skin symptoms (20). In this circumstance, the initiative query of psoriasis family history and physical examinations of skin and nails might reduce the missed diagnosis of PsA. Thus, we investigated the practice habits of rheumatologists in responding to key clues of PsA. When patients showed some specific joint manifestations like seronegative peripheral arthritis, asymmetric sacroiliitis, sacroiliitis with negative HLA-B27 and dactylitis, most of rheumatologists would consider the PsA diagnosis and initially ask for psoriasis family history and check skin and nail of patients, indicating that most of rheumatologists agree that inquiry for the medical history and physical examinations are needed when patients show these clinical features. On the other hand, this survey was conducted under real names, some of the respondents may have overreported good habits for the consideration of fame.

The identification of psoriatic skin and nail lesions especially for atypical or hidden psoriasis trapped most rheumatologists in PsA diagnosis in result to consultation with dermatologists. Quite a lot of rheumatologists had no idea of psoriatic nail lesions like leukonychia, oil-drop discoloration, splinter hemorrhages and red spot in the lunula. This indicates the importance of multidisciplinary collaboration, and there is a need for the training of rheumatologists on identification of psoriatic skin and nail lesions.

Diversities in musculoskeletal manifestations are also one of difficulties in PsA diagnosis. Inflammatory arthritis was fundamental for the diagnosis of PsA. When patients with psoriasis developed arthralgia, the diagnosis of PsA should be considered, but differential diagnosis was indispensable, such as RA, osteoarthritis (OA), and gout (5). Based on the definition of PsA, a psoriasis patient with non-inflammatory musculoskeletal manifestations should not be diagnosed as PsA. There were some reports that the prevalence of PsA among psoriasis patients were more than 45%, which might include the wrong diagnosis of PsA once the psoriasis patients presented arthralgia (21). In this study, although only a small part of respondents also mistakenly believed that a diagnosis of PsA could be made once psoriasis patients presented arthralgia, this indicates that not all the rheumatologists are familiar with the concept of PsA. Therefore, it is still important to improve the rheumatologists’ knowledge about PsA to avoid misdiagnosis.

Dactylitis, also called “sausage”-shaped fingers or toes, occurs in almost 50% of PsA patients, and has a prognostic value for PsA progression (22). More radiological damages were detected in digits with dactylitis than those without (23). Therefore, perception of dactylitis is important in PsA diagnosis. Dactylitis is “uniform swelling such that the soft tissues between the metacarpophalangeal and proximal interphalangeal, proximal and distal interphalangeal, and/or distal interphalangeal joint and digital tuft are diffusely swollen to the extent that the actual joint swelling could no longer be independently recognized” (24). Except for the swelling of whole digit, the digital swelling that only affect PIP but not the MCP and DIP joints (**Figures 5B, C, E**) is also termed dactylitis which might have the same pathophysiology (22, 24, 25). However, in CASPAR criteria dactylitis was defined as swelling of an entire digit, which lead to misconception for some rheumatologists (26). Consequently, some atypical dactylitis might be missed. In this survey, only less than half of rheumatologists knew about different types of dactylitis. Therefore, training about dactylitis to make more rheumatologists recognized with dactylitis and have acquaintance of its role in PsA diagnosis is required.

RF and ACPA are diagnostic markers for RA. The seronegative of RF and ACPA are regarded as one of clinical features of PsA to differentiate with RA, which were also included in CASPAR criteria as one item (26). However, the seropositivity of RF/ACPA were found in 5-20% of PsA patients (27–29). Therefore, the seropositivity of RF/ACPA does not exclude a diagnosis of PsA, although nearly thirty percent of rheumatologists have a wrong understanding of this phenomenon. In fact, there is no reliable diagnostic markers for PsA (30), so a large part of respondents regarded ‘absence of biomarkers’ as a difficulty in PsA diagnosis. Although PsA is a kind of inflammatory arthritis, the elevations of acute phase reactants are found in only half of patients with active PsA (31). Thus, not all patients with active PsA will show elevations of acute phase reactants, much less in those with inactive PsA. Therefore the elevations of acute phase reactants should not be a fundamental element in PsA diagnosis. However, a quarter of rheumatologists had misunderstanding on this.

The perceptions of Chinese rheumatologists on PsA diagnosis were investigated and compared in this survey. In general, there was no difference in the perceptions between TCM- and WM-rheumatologists. Specifically, the incidences of misconceptions on inflammatory arthropathy, acute phase reactant, and rheumatoid factor were higher among rheumatologists with older age, working in secondary hospitals and part-time rheumatologists, indicating that these parts of rheumatologists are the subjects that need to be trained emphatically. Several approaches are accessed to improve the perceptions of rheumatologists on PsA diagnosis, such as conducting continuing education courses about PsA, establishing the PsA associations to hold more academic meetings, or creating WeChat public accounts to spread knowledge of PsA.

This survey investigated the perceptions of Chinese rheumatologists on diagnosis of PsA for the first time. However, there are still several limitations. First, there was no time constraint in answering the questionnaire. During browsing the questionnaire, respondents can access information or communicate with other rheumatologists resulting in failure to reflect their real perceptions. Second, this survey adopted non-probability sampling methods and the sample was not taken randomly. Therefore, the representativeness of the samples for the target population is a limitation. There might be reporting bias in the survey since some rheumatologists with poor knowledge of PsA might be unwilling to participate in this survey. Third, there were less respondents from western China, where economic status is not as good as eastern China, resulting in selection bias. Forth, validation of this questionnaire performed in a very small sample (n=20) of rheumatologists. Fifth, several factors may threat the external validity of the results. The samples were not selected at random, therefore the samples might not be representative of the whole target population. In addition, there was possibility that some rheumatologists may deliberately report good diagnosis habits in their clinical practice for the consideration of fame.

In summary, about three quarters of Chinese rheumatologists know about the elements in PsA diagnosis and have good practice habits in diagnosing PsA. Four main challenges in making PsA diagnosis are revealed. In diagnosing PsA, a small part of participants had incorrect knowledge of inflammatory arthropathy, acute phase reactant, and rheumatoid factor. There were no significant differences in the knowledge of PsA and practice habits in diagnosing PsA between WM- and TCM-rheumatologists. The part-time rheumatologists were not as good as full-time rheumatologists in diagnosing PsA.

## Data Availability Statement

The original contributions presented in the study are included in the article/**Supplementary Material**. Further inquiries can be directed to the corresponding author.

## Ethics Statement

The studies involving human participants were reviewed and approved by Ethics Committee of Shanghai Jiao Tong University Affiliated Sixth People’s Hospital, Shanghai, China. The patients/participants provided their written informed consent to participate in this study.

## Author Contributions

S-MD conceived the study. All authors were involved in study design and questionnaire tests. MC collected, analysed, and interpreted the data. MC and S-MD were involved in drafting and revising the manuscript. All authors contributed to the article and approved the submitted version.

## Funding

This study was supported by National Natural Science Foundation of China (No. 81900795 and 82071809).

## Conflict of Interest

The authors declare that the research was conducted in the absence of any commercial or financial relationships that could be construed as a potential conflict of interest.

## Publisher’s Note

All claims expressed in this article are solely those of the authors and do not necessarily represent those of their affiliated organizations, or those of the publisher, the editors and the reviewers. Any product that may be evaluated in this article, or claim that may be made by its manufacturer, is not guaranteed or endorsed by the publisher.
